# Failure of a Single-Session Intralesional Triamcinolone Injection to Prevent a Stricture After ≥90% Circumferential Rectal Endoscopic Submucosal Dissection: A Case Report

**DOI:** 10.7759/cureus.105100

**Published:** 2026-03-12

**Authors:** Taiji Yoshimoto, Ayaka Nakanishi, Hiroshi Takihara

**Affiliations:** 1 Department of Gastroenterology, Tachikawa Sogo Hospital, Tachikawa, JPN; 2 Department of Gastroenterology, Kishiwada Tokushukai Hospital, Kishiwada, JPN; 3 Department of Gastroenterology, Uji Tokushukai Hospital, Uji, JPN

**Keywords:** circumferential defect, colorectal endoscopic submucosal dissection, postoperative rectal stricture, prophylactic intralesional steroid injection, steroid prophylaxis

## Abstract

Near-circumferential colorectal endoscopic submucosal dissection (ESD) carries a substantial risk of postoperative strictures, with reported rates of 11-43% when mucosal defects involve ≥90% of the luminal circumference and up to 50-71% after total circumferential resection. A postoperative stricture sometimes requires repeated interventions, such as endoscopic balloon dilation, which can impose a significant burden on both patients and healthcare providers. Although an intralesional triamcinolone acetonide (TAC) injection is widely used to prevent strictures after esophageal ESD, its efficacy after colorectal ESD remains unclear. We describe a case of near-circumferential rectal ESD in which prophylactic intralesional TAC injection was administered, yet a postoperative rectal stricture still developed and required endoscopic balloon dilation. A 70 mm type 0-Is + IIa lesion (Paris classification) located in the upper rectum, involving more than 90% of the luminal circumference, was successfully resected en bloc by ESD. Prophylactic intralesional TAC (50 mg) was injected into the post-ESD ulcer base immediately after resection. The postoperative course was uneventful. The patient had no symptoms at the one-month postoperative follow-up visit. At that time, a follow-up colonoscopy was scheduled for approximately six weeks later. However, constipation developed approximately six weeks after ESD and was initially managed with laxatives prescribed by another physician at our hospital. As the constipation worsened, the patient revisited our department, and a colonoscopy was performed the following day, revealing a rectal stricture. Endoscopic balloon dilation was subsequently performed, resulting in symptomatic and endoscopic improvement. A rectal stricture can still develop after near-circumferential colorectal ESD despite prophylactic intralesional TAC injection. These findings highlight the limitations of a single-session intralesional TAC injection and underscore the need for optimized preventive strategies and careful postoperative surveillance in high-risk colorectal ESD involving more than 90% of the luminal circumference.

## Introduction

Endoscopic submucosal dissection (ESD) enables en bloc resection of large colorectal neoplasms; however, lesions with near-circumferential or circumferential extension remain technically demanding and are associated with postoperative complications, particularly stricture formation. Previous studies have demonstrated that mucosal defects involving more than 90% of the luminal circumference are strongly associated with postoperative stenosis after colorectal ESD [1-5]. Postoperative stricture sometimes requires repeated interventions, such as endoscopic balloon dilation, which can impose a significant burden on both patients and healthcare providers. Although an intralesional triamcinolone acetonide (TAC) injection has been shown to be effective in preventing a stricture after esophageal ESD [6,7], its efficacy in colorectal ESD remains uncertain because of limited supporting evidence. A single-session intralesional TAC injection has been reported to successfully prevent strictures in some cases of colorectal ESD [8]. However, some retrospective studies have not demonstrated a significant reduction in stricture incidence with steroid injection [4,5]. Therefore, the preventive efficacy of steroid injection in colorectal ESD remains unclear [9,10]. Moreover, reports specifically documenting the failure of prophylactic intralesional TAC injection in near-circumferential colorectal ESD remain scarce.

Herein, we report a case of near-circumferential rectal ESD in which a prophylactic intralesional TAC injection was administered, yet a postoperative rectal stricture developed, requiring endoscopic balloon dilation.

## Case presentation

**Figure 1:**
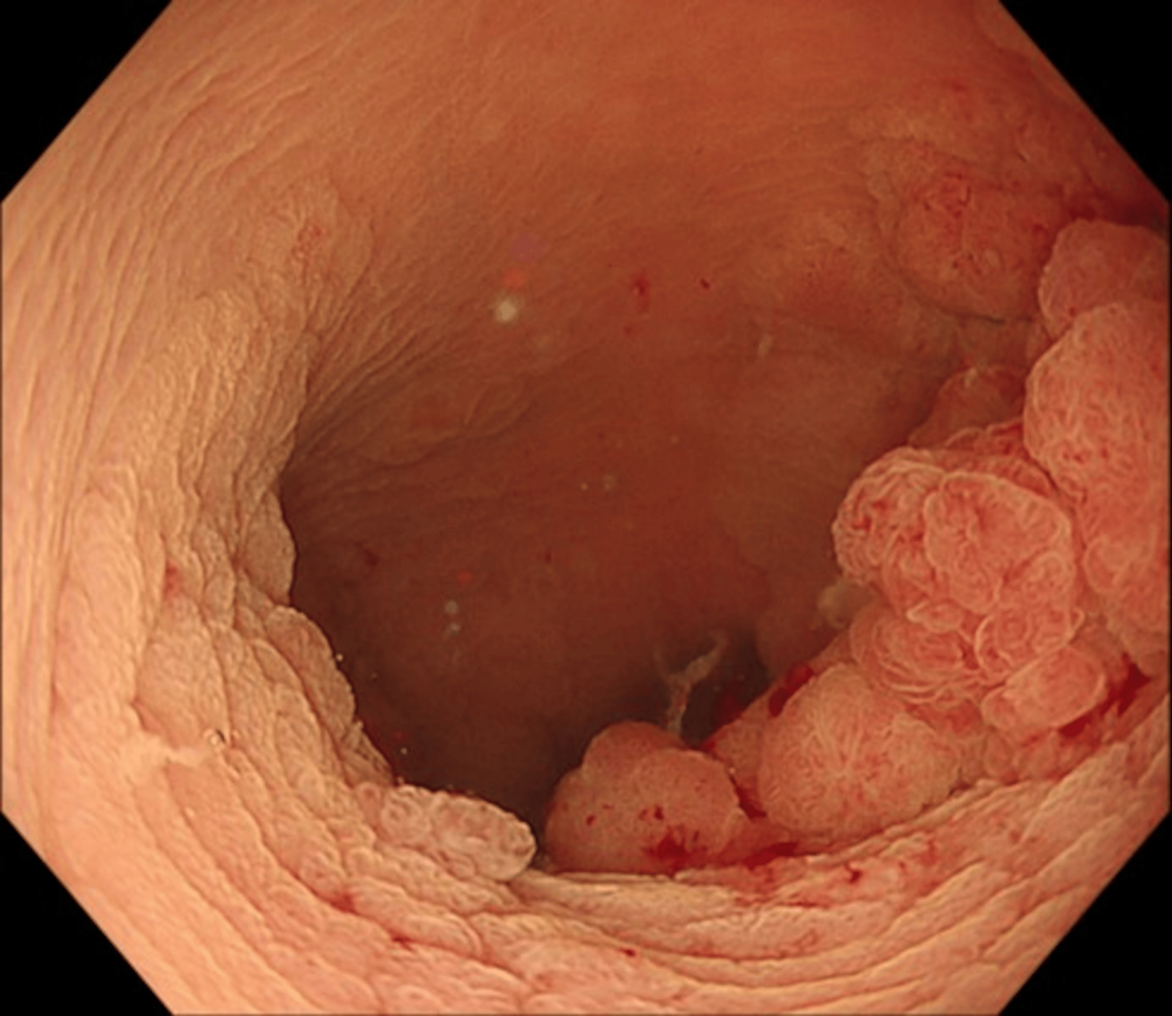
Colonoscopic view showing a 70 mm type 0–Is + IIa lesion (Paris classification) in the upper rectum with near-circumferential extension. Paris classification [11]

A man in his 70s underwent colonoscopy following a positive fecal immunochemical test, which revealed a type 0-Is + IIa lesion (Paris classification [11]) approximately 70 mm in diameter located in the upper rectum, involving nearly the entire luminal circumference (Figure 1).

**Figure 2:**
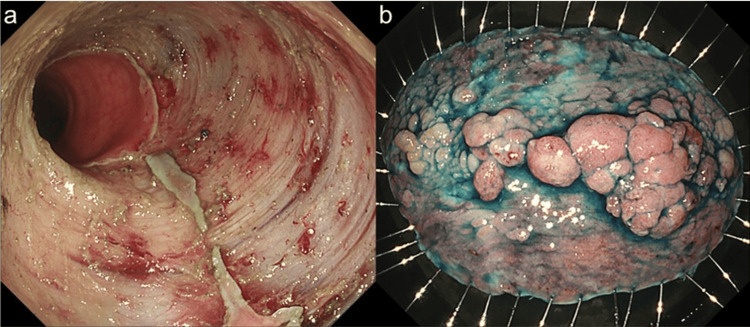
(a) Post-ESD ulcer base demonstrating a near-circumferential mucosal defect involving more than 90% of the rectal lumen. (b) En bloc resected specimen measuring 77 × 53 mm.

ESD was performed under conscious sedation using the PCF-H290TI (Olympus Medical Systems Co., Tokyo, Japan) with a transparent distal attachment. Circumferential marking, mucosal incision, and submucosal dissection were performed using a Proknife 2.0 mm (ORISE ProKnife; Boston Scientific, MA, USA). The pocket-creation method was applied during the procedure. En bloc resection was achieved without perforation. Procedure time was 140 minutes, and the resected specimen measured 77 × 53 mm (Figure 2).

Prophylactic intralesional triamcinolone injection

**Figure 3:**
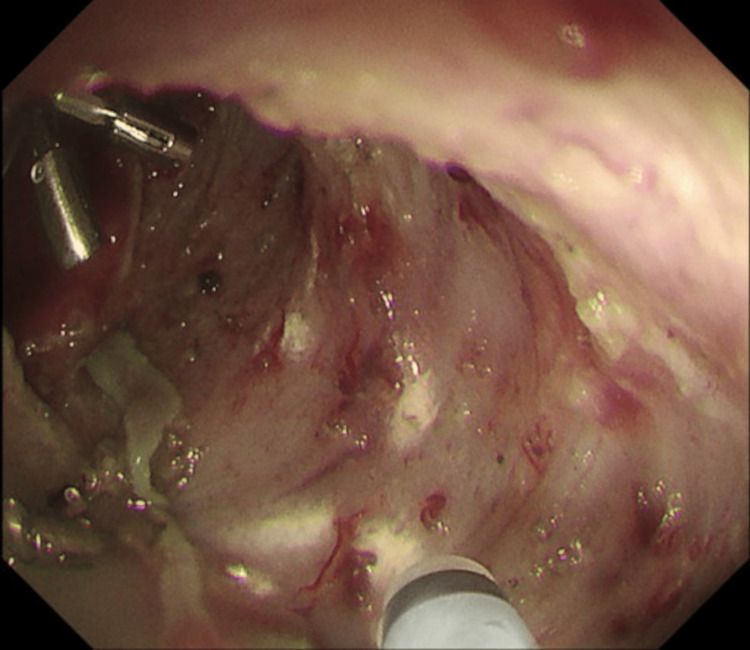
Prophylactic intralesional triamcinolone injection into the post-ESD ulcer base immediately after en bloc resection ESD: endoscopic submucosal dissection

Given the near-circumferential mucosal defect and high risk of postoperative stricture, intralesional TAC was administered immediately after ESD. A total of 50 mg TAC (Kenacort®, 10 mg/mL) was diluted with 0.9% NaCl to a final concentration of 5 mg/mL. Subsequently, 0.5 mL aliquots were injected at multiple points evenly distributed across the entire ulcer base using a 26-gauge, 3 mm needle, with the needle tip kept within the sheath and the sheath gently pressed against the ulcer base to avoid injection into the muscularis propria (Figure 3).

Postoperative course

**Figure 4:**
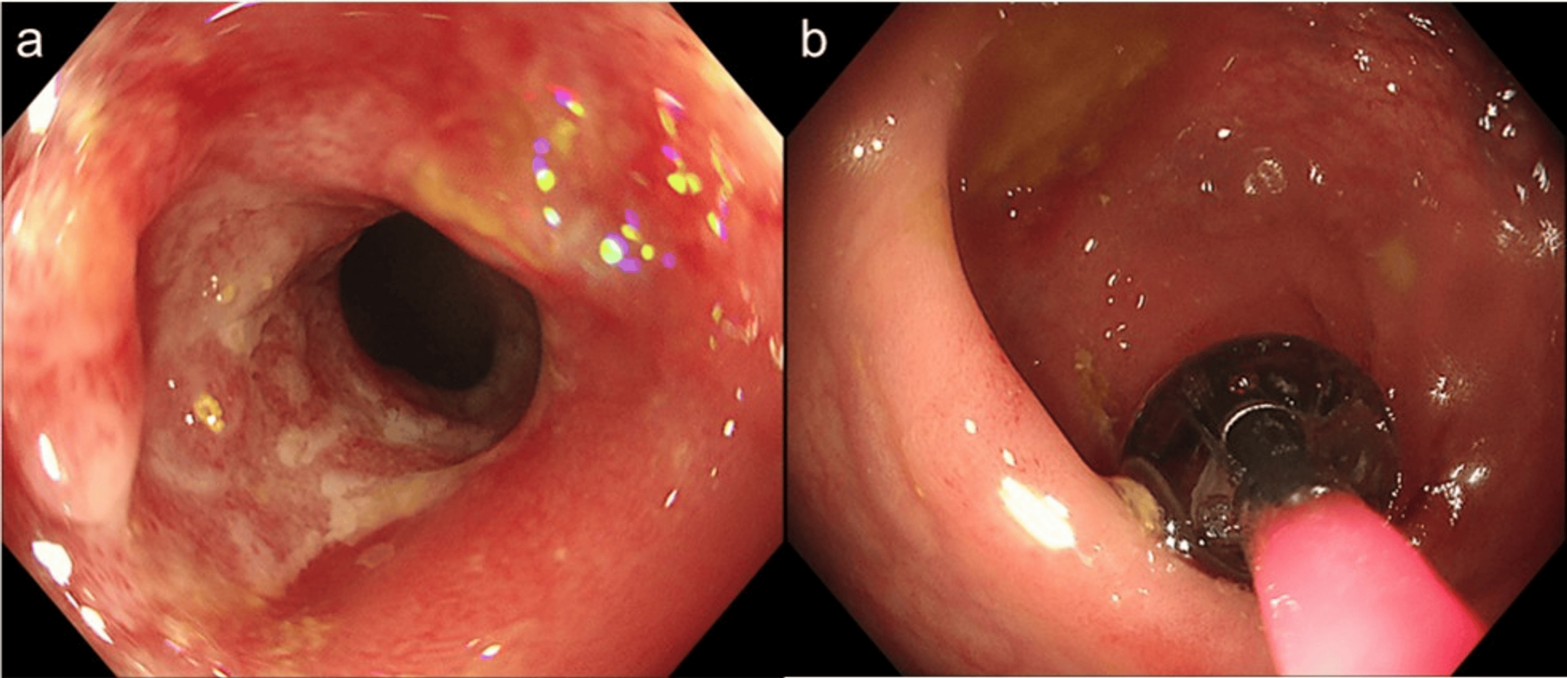
(a) Follow-up colonoscopy showing a rectal stricture with luminal narrowing preventing scope passage. (b) Endoscopic balloon dilation was performed for the post-ESD rectal stricture, resulting in successful luminal expansion. ESD: endoscopic submucosal dissection

The postoperative course was uneventful, and the patient was discharged on postoperative day 6. The patient had no symptoms at the one-month postoperative follow-up visit. At that time, a follow-up colonoscopy was scheduled for approximately six weeks later. However, constipation developed approximately six weeks after ESD and was initially managed with laxatives prescribed by another physician at our hospital. As the constipation worsened, the patient revisited our department, and a colonoscopy was performed the following day, revealing a rectal stricture that prevented the passage of a standard colonoscope. Endoscopic balloon dilation (4 atm, 12 mm) was performed, resulting in symptomatic and endoscopic improvement (Figure 4).

Histopathology revealed an intramucosal, well-differentiated adenocarcinoma arising in the tubular adenoma. The en bloc resected specimen measured 77 × 53 mm, and both horizontal and vertical margins were negative.

## Discussion

Near-circumferential colorectal ESD is technically demanding and is associated with a high risk of postoperative stricture. Previous studies have demonstrated that mucosal defects involving more than 90% of the luminal circumference are strongly associated with stenosis after colorectal ESD. Reported stricture rates range from 11.1-43.8% when defects involve 90% to <100% of the circumference and increase to 50-71.4% after total circumferential resections [1-5]. Anatomical factors may also influence stricture formation. Sako et al. demonstrated that ESD involving the anal canal significantly increases postoperative stricture risk due to limited tissue compliance and functional constraints [4]. However, the present lesion did not involve the anal canal, suggesting that the extensive circumferential mucosal defect itself was the principal contributor to postoperative stenosis in this case.

Unlike esophageal ESD, where an intralesional TAC injection is supported by prospective studies and widely accepted as standard preventive therapy [6,7], its role after colorectal ESD remains unclear [9,10]. A previous case report described successful prevention of stricture after near-circumferential colorectal ESD using a single-session intralesional TAC injection (40 mg) [8]. Based on this report demonstrating successful prevention with single-session intralesional TAC injection, we adopted the same strategy in the present case; however, the stricture still developed. Moreover, some retrospective studies have not demonstrated a significant reduction in stricture incidence with steroid injection alone [4,5]. Therefore, the failure observed in this case cannot be explained solely by the steroid dose.

In esophageal ESD, extensive mucosal defects are well-recognized as a major risk factor for postoperative stricture. Previous studies have shown that local steroid injection alone may be insufficient in such cases, and combination strategies, including intralesional TAC injection combined with short-term oral steroid therapy, have been introduced to prevent stricture formation [10,12]. These findings suggest that similar combination strategies may also be necessary for colorectal ESD, particularly in cases involving mucosal defects exceeding 90% of the luminal circumference. Prospective studies are required to clarify the optimal preventive strategy for near-circumferential colorectal ESD.

Early detection and management of postoperative stenosis are essential. According to recent data, most post-colorectal ESD stenoses can be managed endoscopically without the need for surgery [5]. In esophageal ESD, post-procedural strictures typically develop within 2-4 weeks, with reported median onset times of approximately 15-18 days. In contrast, strictures after colorectal ESD generally occur later, with a reported median time to symptom onset of 34 days (IQR 23-90 days) [5]. In the present case, constipation developed approximately six weeks after ESD, which is within the reported range. This delayed onset of stricture after colorectal ESD, compared with esophageal ESD, may be partly explained by the anatomical and physiological characteristics of the rectum. Repeated luminal distension caused by solid stool retention and passage may exert a mechanical dilating effect on the lumen, potentially delaying the progression of symptomatic stenosis compared with the esophagus.

This case underscores the importance of proactive surveillance and early endoscopic evaluation when obstructive symptoms occur following high-risk colorectal ESD, even when prophylactic intralesional steroid injection has been administered. Careful follow-up within four weeks and for up to three months after near-circumferential colorectal ESD may be necessary to facilitate detection of luminal narrowing before the onset of clinically significant obstructive symptoms.

## Conclusions

This case suggests that a single-session intralesional TAC injection may be insufficient to prevent stricture after ≥90% circumferential rectal ESD. Extensive mucosal defects involving nearly the entire rectal circumference represent a very high-risk situation for post-ESD luminal narrowing. More intensive preventive strategies, such as intralesional TAC injections combined with oral steroid therapy and careful follow-up within four weeks and for up to three months, may be considered in such cases.
